# Activation of STING Based on Its Structural Features

**DOI:** 10.3389/fimmu.2022.808607

**Published:** 2022-07-19

**Authors:** Behzad Hussain, Yufeng Xie, Uzma Jabeen, Defen Lu, Bo Yang, Changxin Wu, Guijun Shang

**Affiliations:** ^1^ The Key Laboratory of Medical Molecular Cell Biology of Shanxi Province, The Institutes of Biomedical Sciences, Shanxi University, Taiyuan, China; ^2^ Department of Basic Medical Sciences, School of Medicine, Tsinghua University, Beijing, China; ^3^ Institute of Microbiology, University of Veterinary and Animal Sciences, Lahore, Pakistan; ^4^ CAS Key Laboratory of Pathogenic Microbiology and Immunology, Institute of Microbiology, Chinese Academy of Sciences, Beijing, China; ^5^ Shanxi Provincial Key Laboratory of Protein Structure Determination, Shanxi Academy of Advanced Research and Innovation, Taiyuan, China; ^6^ Shanxi Provincial Key Laboratory for Major Infectious Disease Response, Shanxi Academy of Advanced Research and Innovation, Taiyuan, China

**Keywords:** stimulator of interferon genes, cyclic GMP-AMP, cyclic GMP-AMP Synthase (cGAS), innate immunity, cyclic dinucleotide

## Abstract

The cGAS-cGAMP-STING pathway is an important innate immune signaling cascade responsible for the sensing of abnormal cytosolic double-stranded DNA (dsDNA), which is a hallmark of infection or cancers. Recently, tremendous progress has been made in the understanding of the STING activation mechanism from various aspects. In this review, the molecular mechanism of activation of STING protein based on its structural features is briefly discussed. The underlying molecular mechanism of STING activation will enable us to develop novel therapeutics to treat STING-associated diseases and understand how STING has evolved to eliminate infection and maintain immune homeostasis in innate immunity.

## Introduction

**Figure 1:**
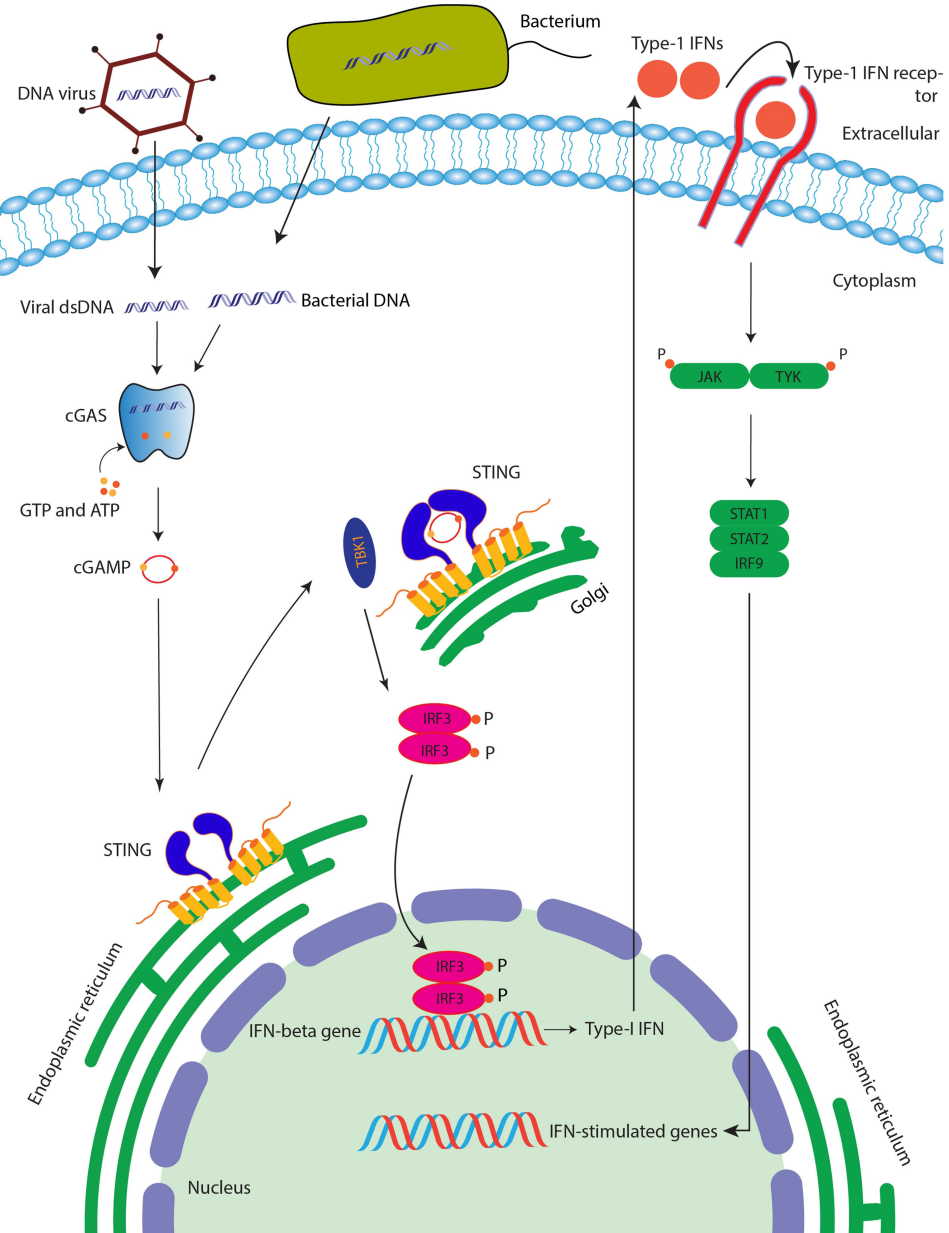
STING signaling pathway. After binding with the cGAMP (which is produced after the cGAS enzyme senses the double-stranded DNA), STING protein is activated followed by the activation of TBK1, which then phosphorylates transcription factor IRF3, which is then translocated into the nucleus of the cell leading to induction of interferon genes and ultimately anti-viral and anti-cancer immunity.

Exogenous dsDNA in the cytosol, such as viral infection, is a danger signal sensed by the innate immune system and triggers immune responses (1). Stimulator of interferon genes (STING), an endoplasmic reticulum adaptor protein (also known as ERIS, MPYS, MITA, and TMEM173), links the cytosolic detection of dsDNA to the type I interferon (IFN) signaling and elicits a rigorous innate antiviral immune response (2–6). In this pathway, cytosolic DNA but not RNA induces cyclic GMP-AMP synthase (cGAS) to synthesize metazoan second messenger 2′3′- cyclic GMP-AMP (2′3′-cGAMP) with ATP and GTP as substrate (7–10). STING, observed as an obligate homodimer, binds to 2′3′-cGAMP or other cyclic di-nucleotides (CDNs) produced by bacteria and undergoes a series of conformational changes (11–25). Activated STING then traffics to Golgi, where it recruits and activates TANK Binding Kinase 1 (TBK1) and transcription factor IRF3. Activated IRF3 dimerizes and enters the nucleus, promoting, the expression of type I interferon as well as activating the nuclear factor κ-light chain enhancer of activated B cells (NF-κB) pathway (2–5, 26–28) (**Figure 1**). However, if STING is activated in an uncontrolled manner, it will cause autoimmune diseases such as Aicardi–Goutieres syndrome (AGS) (29) and STING-associated vasculopathy with onset in infancy (SAVI) (30). In recent years, STING activation, in combination with immune checkpoint blockade (ICD), was also found to be essential for the success of tumor immunotherapy (31). Considering the importance of STING in anti-infection, autoimmune diseases, and anti-tumor therapy, it is necessary to figure out the STING working mechanism from a structural insight at the atomic level. Here, we summarized the recent studies of apo and ligand-bound STING structures, oligomerization of STING, as well as the disease-linked STING mutations from a structural viewpoint.

## The Architecture of Apo Sting

**Figure 2:**
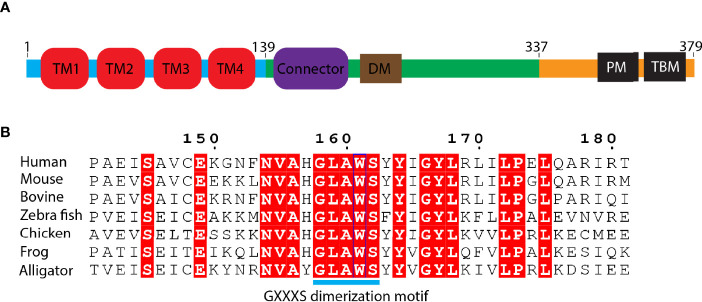
Domain organization of STING protein. **(A)** Schematic representation of human STING domain architecture. Blue bar: N-terminal domain; green bar: ligand binding domain (LBD); orange bar: C-terminal tail (CTT). DM, dimerization motif. PM, phosphorylation motif. TBM, TBK1 binding motif. **(B)** Sequence alignment of the region of LBDα1 from various species. The number is based on human STING. GXXXS motif is labeled as cyan bar.

**Figure 3:**
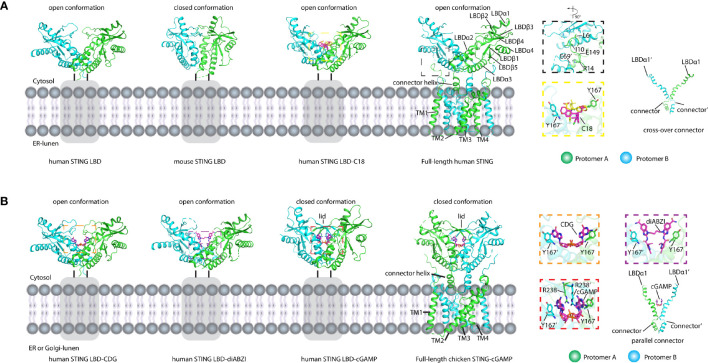
Structures of LBD domain and full-length STING in apo and ligand-bound forms. **(A)** Structures of apo and antagonist bound LBD and apo full-length STING shown in cartoon. The secondary structures referred to in the text were labeled. The residues and compounds are shown as sticks. Gray rectangles represent TM domain. The black dashed square shows the magnified view of interactions between LBD and the N-terminal segment. The yellow dashed square shows the magnified view of LBD bound with antagonist C18. The connectors cross over each other. PDB IDs: 4F5W (human STING LBD), 4KC0 (mouse STING LBD), 6MXE (human STING LBD-C18), and 6NT5 (full-length human STING). **(B)** Structures of agonist bound LBD and full-length STING shown in cartoon. The binding of cGAMP induced the closed conformation and the 180° rotation of the LBD domain relative to the TM domain. The orange dashed square shows the magnified view of c-di-GMP (CDG) bound to STING LBD. The purple dashed square shows the magnified view of diABZI bound to LBD. The red dashed square shows the magnified view of cGAMP bound to LBD. The connectors are parallel to each other. The secondary structures referred to in the text were labeled. The residues and compounds were shown as stick. Gray rectangles represent the TM domain. PDB IDs: 4F5Y (human STING LBD-CDG), 6DXL (human STING LBD-diABZI), 4KSY (human STING LBD-cGAMP), and 6NT7 (full-length chicken STING–cGAMP).

STING is an endoplasmic reticulum membrane protein (3) that contains an N-terminal transmembrane domain (TMD) with four transmembrane helices (residues 1–138) (14), a cytosolic ligand-binding domain (LBD, residues 139–336), and a C-terminal tail (CTT, residues 337–379) (residue number is based on human STING) (**Figure 2A**). Before the determination of the STING structure, the domain boundary of STING was unclear, and the topology of STING was controversial. It was thought that there were five putative TM helices in the TMD by bioinformatics prediction (2, 32), which raises the question of the cellular position of the LBD domain (in ER lumen or cytosol). The subsequent structural studies of the LBD domain show that the fifth helix takes part in the formation of the dimer interface (**Figure 2B**), which clarified the composition of the TMD domain with four helices and the cytosolic location of the LBD domain. The cytosolic face of the LBD was further confirmed by an immuno-electron microscopy (EM) study of STING-GFP vesicle (33). The crystal structure of the apo STING LBD domain solved by several groups generated numerous 3D information to understand its related biochemical properties (11, 13, 16–19, 21, 34). The crystal structure of the LBD of human STING shows that the LBD forms a constitutive dimer, which is consistent with the result measured by dynamic light scattering (DLS) (34). The overall architecture of the LBD shows a butterfly-like structure with each protomer as its wing and a crevice formed between the two protomers (**Figure 3A**). The protomers adopt an α+β fold consisting of a five-stranded sheet in the center and four helices in periphery. The first long kinked helix (LBDα1) mediates the dimer interface formation, and residues in this helix are highly conserved. Interestingly, there is a dimerization motif GXXXS (X denotes any residue) at the interface (**Figure 2B**), which is usually found in the transmembrane helix mediating their lateral associations and signal transduction (35). Apparently, the dimerization motif plays a vital role in guiding the folding of STING protein, as demonstrated by the mutagenesis study (11) and the transduction of the signal to the TMD domain (14). The loop region between LBDβ2 and LBDβ3 is invisible in the human apo STING structure, whereas it is ordered and adopts various conformations in mouse, sea anemone, rat, and fly STING LBD (**Figure 3A**). Besides the LBDβ2–β3 loop region, the distances between the tips of the two protomers vary among these species, which raises the concern of crystal packing artifact of apo STING LBD. The CTT conformation in all solved structures has never been determined, although several studies claim that the CTT could bind to the other part of the LBD, playing a role in STING autoinhibition (13, 23, 36). In contrast to the in-depth structural studies of STING LBD, the knowledge about TMD is scarce owing to the challenging property of the full-length protein. With the development of cryo-EM in recent years, the full-length structures of human and chicken STING were solved at near-atomic resolution, providing this field a panoramic view of the STING molecule (14) (**Figure 3A**). Through structural comparison, LBDs of full-length human and chicken STING are almost the same as the crystal structure of the LBDs from human and rat. Like the LBD, the TMD also dimerizes, resulting in a domain-swapped dimer of full-length STING. As for the TMD, the TM2 and TM4 are located at the center, while the TM1 and TM3 are at the periphery of the helical bundle. There are some extra structural features observed in the full-length structure. One prominent characteristic is the formation of two amphibian helices between the LBD and TMD. These two helices, known as the connector helix, adopt right-handed coiled-coil conformation, and the connector loop linking the connector helix and the LBD crosses over each other (**Figure 3A**). Another feature is the interaction between the N-terminal segment from one protomer and the LBD from the other protomer. Two conserved hydrophobic residues (L6 and I10) within the N-terminus plug into the hydrophobic pocket outside the LBD, and R14 engages E69 and E149 from TM2 and the connector helix, respectively (**Figure 3A**). Mutagenesis studies demonstrated that these residues play a crucial role in STING folding (14, 16).

## Ligand Recognition and Signal Transduction

It has long been a mystery before discovering the endogenous ligand of STING. In 2011, cyclic di-GMP (c-di-GMP), the second messenger in Gram-negative bacteria, was identified as a pathogen-associated molecular pattern (PAMP) to directly bind to and activate mouse STING (32). After that, the studies on how STING recognizes cyclic di-nucleotide (CDN) were performed rigorously. Five groups reported the c-di-GMP/human STING-LBD complex (11, 13, 17, 18, 34). All of the studies show that the c-di-GMP is located at the dimer interface (**Figure 3B**). Four of them show that the c-di-GMP binding does not induce any conformational changes (11, 13, 17, 34) and the recognition of c-di-GMP is largely mediated by the π–π stack between the purine ring and Tyr167 in LBDα1 and solvent-mediated hydrogen bonds (34) (**Figure 3B**). The weak interactions between human STING and c-di-GMP are consistent with the function studies, demonstrating that the c-di-GMP is not a good stimulator of human STING (17, 23). In 2013, with the discovery of the cGAS enzyme, its product 2′3′-cGAMP was identified as STING’s endogenous ligand with a much higher binding affinity (*K*_d_ ~nM) than that of the c-di-GMP (*K*_d_ ~μM) (7–9, 12, 15, 37). The subsequent structural studies of the 2′3′-cGAMP/STING complex provide more information about the STING activation (12, 15). Compared with c-di-GMP, 2′3′-cGAMP induces huge conformational changes, and the conformation of STING in complex with 2′3′-cGAMP is largely different from that of the apo STING **(** **Figure 3B** **)**. First, the two protomers in the complex undergo inward rotations relative to the twofold axis, creating a deeper crevice between the two protomers. Second, LBDβ2–β3 loops from two protomers move close to each other, forming a lid of a four-stranded antiparallel β sheet, which is disordered in the apo state. Therefore, the 2′3′-cGAMP-bound STING is in the closed state because of the lid formation of two protomers, while apo STING in the absence of this lid is in an open conformation. These 2′3′-cGAMP-induced conformational changes were observed not only in human STING, but also in mouse, rat, sea anemone, pig, and fly (12, 21, 24, 38), which is in big contrast to the species specificity observed in c-di-GMP binding. Besides the stacking interaction occurring between the purine and the Tyr167, other strong and extensive interactions can be observed in the 2′3′-cGAMP-STING complex (**Figure 3B**), such as the charge–charge interactions between the R238 and phosphate group in 2′3′-cGAMP, and its important role in binding was confirmed by the mutagenesis study (15).

After the determination of the 2′3′-cGAMP/full-length chicken STING structure, we can gain more structural information regarding STING activation. 2′3′-cGAMP not only induces the formation of lid but also promotes the 180° clockwise rotation of the LBD relative to the TMD (14). The two crossed connector loops in the apo structure were converted to a parallel configuration in the 2′3′-cGAMP-bound structure (**Figure 3B**). Although the huge conformational rearrangements were observed in the LBD and the connector, there were no significant changes occurring at the TMD part (14).

**Figure 4:**
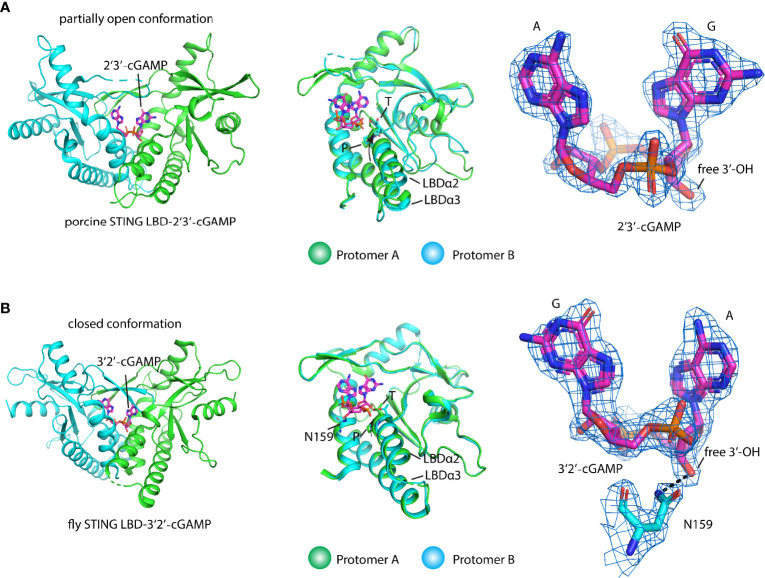
Structural basis of ligand recognition and discrimination by STING. **(A)** Porcine STING LBD engages 2′3′-cGAMP (PDB ID: 6A06) with a partially open conformation (left panel). 2′3′-cGAMP adopts a fixed conformation in contrast to an alternative conformation that exists in other 2′3′-cGAMP-complexes. Superimposition of protomer A and protomer B (middle panel). The TP motif in two protomers, the key ligand recognition and discrimination element, adopts different conformations. The arrow denotes the tilt of the LBDα2–α3 helical bundle in protomer A relative to it in protomer B, 2Fo-Fc electron-density map for 2′3′-cGAMP (blue mesh) is shown and contoured at 2 *σ* (right panel). **(B)** Fly STING LBD-2′3′-cGAMP complex (PDB ID: 7MWZ) in a closed conformation (left panel). 2′3′-cGAMP adopts a fixed conformation in the complex structure. Superimposition of protomer A and protomer B (middle panel). The TP motif in two protomers adopts different conformations. The arrow denotes the tilt of the LBDα2–α3 helical bundle in protomer A relative to it in protomer B. The ligand discrimination residue N was shown. 2Fo-Fc electron-density map for 3′2′-cGAMP (blue mesh) and residue N is shown and contoured at 2 *σ* (right panel). Black dashed line shows the hydrogen bond.

As the endogenous STING ligand, 2′3′-cGAMP is structurally distinct from the bacterial cyclic dinucleotide (CDN) since it contains an unusual 2′-5′ phosphodiester bond linking adenosine and guanosine besides a common 3′-5′ phosphodiester bond linking adenosine and guanosine [Gp(2′-5′)Ap(3′-5′)] (8, 9, 15). One of the long-lasting questions in STING ligand recognition is why STING, a constitutive homodimer, prefers asymmetric ligand 2′3′-cGAMP over symmetric ligand such as 3′3′-CDN. Since STING engages CDN in an almost same closed conformation and the two protomers also adopt the same conformation and the 2′3′-cGAMP in these structures exhibit alternative conformations with two purines switching position, it is hard to interpret this from the structural study of STING from several species, such as human. To answer this question, Shi et al. found that 2′3′-cGAMP, not other isomers such as 3′2′-cGAMP and 2′2′-cGAMP, tends to adopt an organized ligand-free conformation that pays less entropy to engage STING and leads to the highest binding affinity of 2′3′-cGAMP. Based on this idea, locking c-di-GMP in rigid ligand-free conformation *via* introducing a transannular macrocyclic linker between the two purines transformed the c-di-GMP into a pan-genotypic STING agonist (39). One intriguing study on how porcine STING discriminates various CDNs shows that the STING protein itself adopts an asymmetric ligand-binding pocket to bind CDN (24). The two protomers of STING adopt different conformations with a partially open lid region and the shift of the LBDα2–LBDα3 helical bundle in one protomer compared with the other one, which has never been observed in other STING–ligand complex structures (**Figure 4A**). The shape complementary and extensive interactions between porcine STING and 2′3′-cGAMP make it the most potent ligand. At the same time, the symmetric conformation of the other 3′3′-CDN engages the asymmetric binding pocket in STING with bad contacts disfavoring their binding. Of note, the TP motif adopting a different conformation in the two protomers plays an important structural role in the recognition of ribose with 2′-5′ and 3′-5′ linkages (24) (**Figure 4A**). The asymmetric ligand-binding recognition could also be found in the 3′2′-cGAMP-bound fly STING structure in which the conformation of the LBDα2–LBDα3 helical bundle in each protomer resembles the porcine STING–2′3′-cGAMP complex. In addition, it seems that the residue N159 in fly LBD could interact with the free 3′ hydroxyl group in ribose, rendering fly STING to prefer 3′2′-cGAMP to 2′3′-cGAMP (38) (**Figure 4B**). Therefore, both the ligand and the protein itself contribute to the STING ligand discrimination.

**Figure 5:**
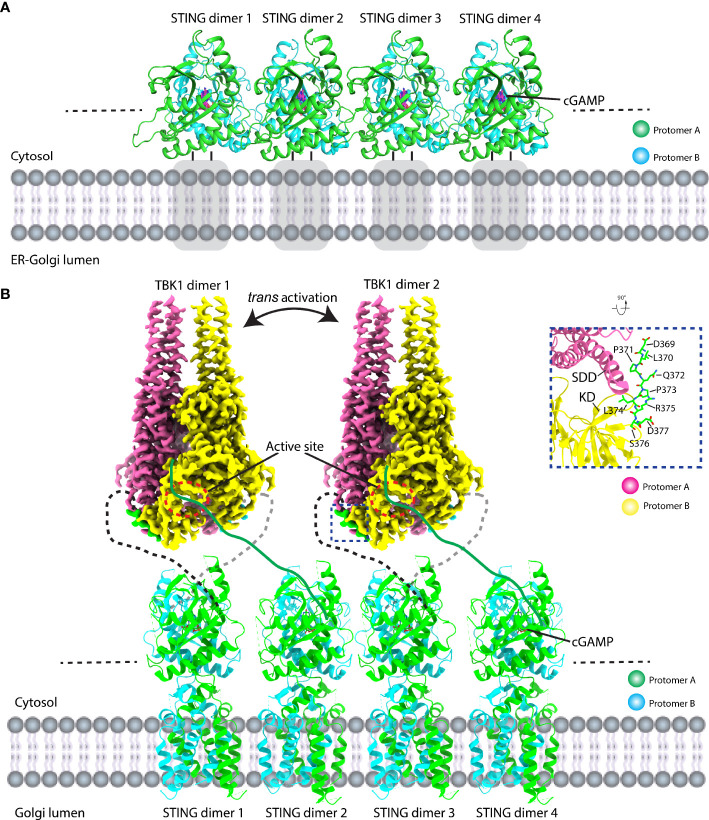
Structural basis of STING signal transduction. **(A)** Crystal packing analysis shows that the ligand-bound LBD forms a linear array of STING dimer in a side-by-side manner in the crystal lattice. PDB ID: 4KSY. Gray square represents the TM domain. **(B)** Structural model of TBK1 binds to and phosphorylates STING CTT. Full-length chicken STING–cGAMP adopts a linear assembly with open ends. The cryo-EM density of the TBK1-CTT complex is shown. TBK1 binds to STING CTT (black dashed line) in a head-to-head manner and phosphorylates CTT from the adjacent STING dimer (CTT: green line). The zoomed-in view of the detailed interaction between TBK1 and CTT is shown (right panel). PDB ID: 6NT8 and 6NT9 for chicken STING–cGAMP tetramer and human TBK1-chicken STING CTT, respectively.

**Figure 6:**
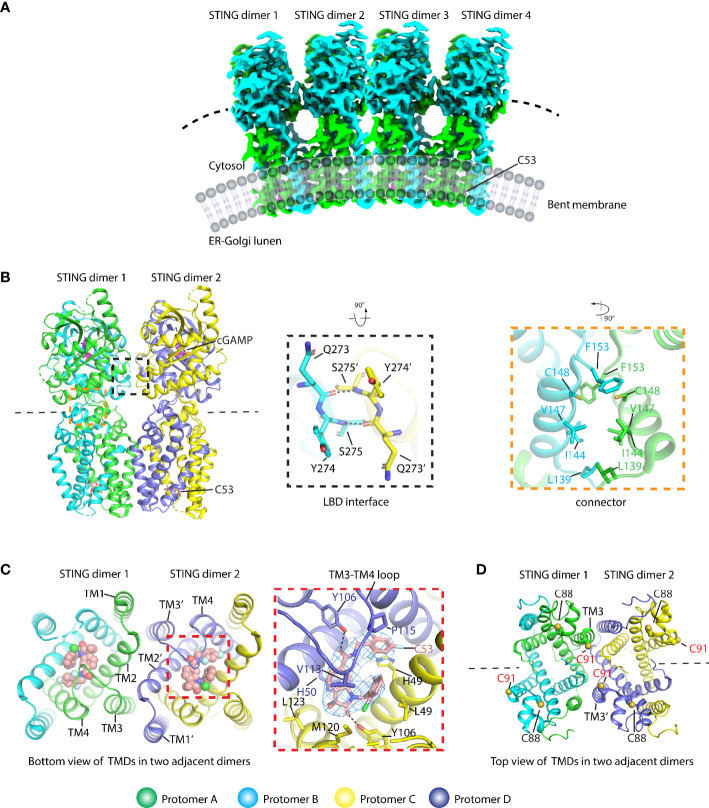
Structural basis of human STING oligomer induced by cGAMP and compound C53 which binds to a novel cryptic pocket in the TM domain (PDB ID: 7SII). **(A)** Cryo-EM density of the human STING/cGAMP/C53 oligomer shows a bent conformation. **(B)** Cartoon representation of the STING tetramer bound with cGAMP and C53 (left panel). The magnified black dashed square shows the interaction between two adjacent LBD domains (middle panel). The magnified orange dashed square shows the interaction network in the connector region. The residues referred to in the text were labeled. Black dashed line shows the hydrogen bond. **(C)** The oligomer interactions were observed in the TM domain (right panel). The detailed interaction between C53 and the STING TM domain (middle panel). The cryo-EM density for C53 contoured at 3 *σ* is shown in blue mesh. The black dashed line shows the hydrogen bond. **(D)** The localization of C88 and C91 in the oligomer structure. C88 is buried in the structure and C91 is located at TM3 and involved in the formation of the oligomer interface. C53 is shown in both sphere and stick modes.

The molecular mechanism of activated STING transducing signal to downstream TBK1 and IRF3 perplexes this field for an extended period. The biochemical study suggests that the STING could be phosphorylated by TBK1 at the conserved pLxIS motif (p, x, and S denote hydrophilic residue, any residue, and phosphorylation site, respectively) followed by recruitment of IRF3 using this as a docking site, and then IRF3 was also phosphorylated at the similar pLxIS motif in itself (40). The phosphorylated pLxIS motif in IRF3 could compete with the docking site and finally leads to the dimerization of IRF3. The structural study of STING-CTT and the IRF3 complex shows that the hydrophobic residues L and I could insert into the shallow pocket in the CTD of IRF3, and phosphorylated residue S engages positively charged residues on the surface of IRF3 (41). However, how activated STING recruits and activates TBK1 was largely unknown before three independent studies of the STING oligomer formation and the binding between TBK1 and STING (14, 23, 42, 43). The native-PAGE result clearly shows ladder-like band formation upon the cGAMP treatment, confirming the assembly of STING triggered by the ligand in the cells (42, 44–46). Intriguingly, the crystal packing analysis shows linear side-by-side assembly of the LBD in an open-ended fashion, which was observed in the crystal lattice of ligand-bound STING (**Figure 5A**) but not found in ligand-free STING, suggesting that this type of STING oligomerization may have physiological relevance and underlie the native gel result (14, 23). Furthermore, the c-di-GMP-bound bacterial STING could form filaments with a side-by-side packing mode indicating the ligand-induced oligomerization of STING is an evolutionarily conserved event (47, 48). In fact, the accompanying cryo-EM study of the chicken STING–cGAMP tetramer structure demonstrated that the STING/cGAMP complex assembles in a side-by-side manner in solution, which is the same as the pattern observed in a crystal lattice, although there are subtle differences. For instance, no direct contact was observed in the chicken STING tetramer interface between the two adjacent dimers either in the LBD domain or in the TM domain (**Figure 5B**). In contrast, backbone interactions between two neighboring LBDα2–α3 loops were seen in the human LBD/cGAMP crystal lattice (14). A recent structural study of the human STING/cGAMP/compound C53 ternary complex shows that both the LBD and TM domain contribute to the oligomer formation (49) (**Figure 6**). In this structure, the adjacent LBD interactions involving backbone interaction occur at the LBDα2–α3 loop consisting of residues Q273, Y274, and S275, which is consistent with the crystal structure observation (**Figure 6B**). The TM domain interaction implicates the TM1, TM2, and TM4 from one STING dimer and TM3 from the neighboring STING dimer (**Figure 6C**). Compound C53 is captured at the hydrophobic pocket formed by two TM2 and two TM4 and covered by the lid formed by a single TM3–TM4 loop. The residues involved in this pocket are L49, H50, Y106, V113, P115, and M120 (**Figure 6C**). Intriguingly, the TM domain interaction transforms the linear assembly into a slightly bent conformation in contrast to the straight assembly observed in the crystal lattice and chicken STING tetramer (**Figure 6A**). However, since the compound C53 binding pocket is located at the TM domain, it cannot exclude the effect of C53 on the TM domain interaction, and it remains unclear whether cGAMP or C53 themselves could induce the formation of this bent conformation. Superimposing the apo LBD into the cGAMP-bound tetrameric structure, the clashes could be seen in the LBDα2–α3 loop region, suggesting that the LBDα2–α3 loop functioned as an inhibitory element and conformational changes induced by the cGAMP release its inhibition (14).

Because the downstream kinase TBK1 adopts a constitutive dimer with their active sites facing away from each other, which needs another TBK1 dimer for its *trans* activation, the necessity of oligomerization of STING for signal transduction seems reasonable. In order to figure out how the STING and TBK1 complex assemble, two structural studies of the STING–TBK1 complex show that STING binds to TBK1 in a head-to-head manner with a 2:2 ratio (**Figure 5B**). A conserved sequence of (D or E)xPxPLR(S or T)D (x denotes any amino acid) (known as TBK1-binding motif: TBM) within the STING CTT sticks into a shallow pocket formed between the SDD from one TBK1 protomer and the KD from the other TBK1 protomer (42, 43) (**Figure 5B**). The important role played by TBM was confirmed by the mutagenesis studies. Interestingly, the TBM immediately follows the phosphorylation site (pLxIS) with only a two-residue distance and the pLxIS motif in TBK1-bound CTT is located far away from the two catalytic centers of the TBK1 dimer, which indicates that the TBK1 could not phosphorylate the CTT once it was bound but could phosphorylate the CTT from the neighboring STING dimer. Therefore, STING oligomerization provides a solution for TBK1 trans-autophosphorylation, TBK1 binding, and STING phosphorylation. Nevertheless, how the IRF3 docks in the STING/TBK1 complex remains obscure and the future direction could be the determination of the supercomplex of STING/TBK1/IRF3.

## STING Activation and Inhibition *via* Various Mechanisms

How STING is activated by its ligands is the key question in this field. As described above, apo STING adopts an open conformation. At the same time, the endogenous ligand cGAMP induces STING, adopting a closed conformation with the lid formation to bury the ligand in the dimer interface. As for the full-length STING, the formation of closed conformation also couples the rotation of the LBD domain and the formation of a parallel array of STING dimer (14). One study found that the compound diABZI (symmetry-linked amidobenzimidazoles) could activate STING and, unexpectedly, keep STING LBD in an open conformation in both crystal and solution (50) (**Figure 3B**). The structural comparison shows that the diABZI-bound structure is the same as the apo STING and the four c-di-GMP-bound STING structures determined in 2012 (**Figure 3B**), which suggests that both the open and the closed conformation represent the active conformation. In addition, due to the lack of full-length diABZI-bound STING structure, it is not sure whether diABZI could induce the conformational changes observed in the structure of cGAMP-bound full-length STING. As mentioned above, the STING agonist compound C53 engages STING at a cryptic pocket in its TM domain. The finding of a novel agonist binding pocket in the TM domain further suggests that the STING could utilize various mechanisms for its activation. The key point to understand the activation mechanism may exist in the knowledge of how STING was inhibited. Several studies proposed that the CTT is the inhibitory element that can bind to the LBD domain and prevents STING activation, while the ligand binding could release the CTT from LBD sequestration and breaks its autoinhibition (13, 23, 36). However, the structural basis for STING autoinhibition involving CTT remains unclear and needs further investigation.

Since the aberrant activation of STING could lead to the autoimmune diseases such as SAVI and AGS, the inhibition of STING by small molecules has been developed rigorously for therapeutic purposes. The inhibition mechanism of STING by these compounds and their corresponding structural basis is diverse. The LBD with open-to-closed conformation changes induced by the ligand binding prompted people to design a compound to keep its open conformation so that it could compete with cGAMP binding, and thus suppress the activation of STING. For example, compound C18 could bind to a dimer crevice and stick STING in an open conformation (51) (**Figure 3A**). However, C18 is a relatively weak antagonist (IC_50_ = 11 μM), inhibiting the production of IFNβ stimulated by cGAMP. Another antagonist, Astin C, a cyclopeptide separated from the medical plant, could bind to the LBD with a higher affinity (*K*_d_ = 53 nM) and thus inhibits the IRF3 binding and subsequent cytokine release (52). The IC_50_s of Astin C inhibiting IFNβ production are within the range of about 3–8 μM for different cell lines used. Virtual screening of the LBD CDN-binding pocket also found that the STING antagonist SN-011 presumably located underneath the LBDβ2–β3 loop could compete with cGAMP binding, preventing the activation of a STING-related immune response (53). The *K*_d_ value measured by surface plasmon resonance (SPR) is about 4 nM and the IC_50_ calculated is 76 nM, which is comparable to that of the cysteine modification drug H-151 (46). However, the detailed structural basis of Astin C affecting IRF3 engagement and the SN-011-bound structure remains unclear and could be the direction for the future studies. In other aspects, the prevention of STING oligomerization is another way to inactivate STING. C91/C88 modification drugs such as H-151 could abolish STING polymer formation, which is a promising drug candidate to treat STING-mediated autoimmune diseases (46).

Besides the STING activity regulation mentioned above, the post-translational modifications (PTMs) of STING have been demonstrated to regulate the STING signaling, among which modification of C88/C91 in human STING is of importance in either enhancing or inhibiting the STING activity (54–56). The palmitoylation of C88/C91 in the human STING was thought to be required for STING activation at the Golgi apparatus (54). In contrast, nitro-alkylations and carbonylation could inhibit the palmitoylation, resulting in the abolition of IFNβ signaling (55, 56). Previous full-length human STING structure analysis indicates that C91 may be the functional palmitoylation site because it is exposed, while C88 is buried in the protein core and may be inaccessible to the modification (14). According to the human STING/cGAMP/C53 oligomer structure (49), C91 is located at the beginning of the TM3, and it takes part in the formation of the polymer interface (**Figure 6D**). Given that the active STING polymer is formed at the ER before it travels to the Golgi apparatus (23), the chance of palmitoylation is low for those C91 residues in the polymer interfaces since they are inaccessible to the Golgi-localized palmitoyl acyltransferase enzymes (e.g., ZDHHC3) (54). However, as for the C91 localized at the ends of the polymer, they are exposed, and their palmitoylation modification may occur. Therefore, the active STING polymer can only be partially palmitoylated in a much lower percentage. The C88/C91 modification by endogenously formed nitro-fatty acids or compound H-151 could block the STING signaling by preventing STING polymerization on the ER and subsequently travel to Golgi stimulated by the cGAMP. Reactive oxygen species (ROS) were also found to modify cysteine residues in the cytosolic domain, such as C148 (human STING), which could be oxidized to negative regulation of the STING activation (57, 58). A previous study proposed that the C148 residue in the human STING can cross-link the active STING dimer into a long polymer through the formation of inter-dimer disulfide bonds, which is required for the activation of the human STING (23). The oxidized C148 was thought to be unable to form a STING polymer mediated by dimer cross-linking. Through structural analysis of the human STING/cGAMP/C53 polymer, C148 was buried in the hydrophobic core in amphipathic connector helices consisting of residues such as L139, I144, and V147, and it particularly packs against F153 (**Figure 6B**), thus playing a role in stabilizing the active conformation. The modification of C148 suppressing signaling is likely due to its destabilizing effects on the STING polymer.

## STING Structure and Its Related Autoimmune Disease

**Figure 7:**
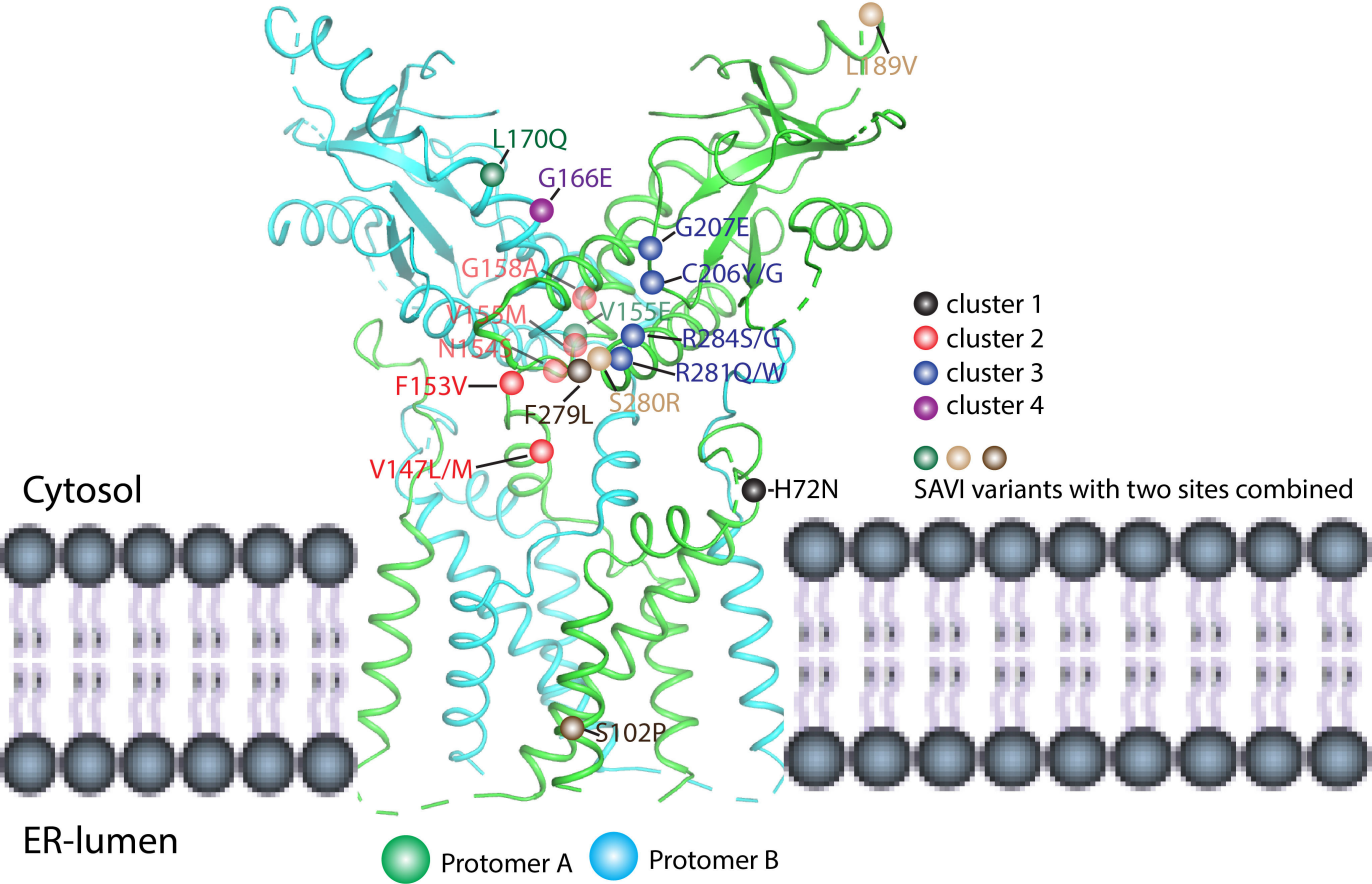
Mapping SAVI mutations in the human STING structure (PDB ID: 6NT5). The SAVI mutations have been located on STING protein and divided into four clusters (1–4). Cluster 1 (black ball) is located at the N-terminal domain and includes the H72N variants. Cluster 2 (red ball) is localized at the dimer interface and shows the V147L/M, F153V, N154S, V155M, and G158A mutations. Cluster 3 (blue ball) is localized at the helical bundle of LBDα2–α3 and the long LBDβ1–β2 loop outside of the dimer interface and includes the C206Y/G, G207E, R281Q/W, and R284G/S mutations. Cluster 4 (purple ball) is located at the ligand-binding pocket and includes the G166E variant. Other than these clusters, there also exist combinations of two mutations; for example, S102P/F279L, V155E/L170Q, and L189V/S280R as shown by brown, green, and light brown balls, respectively.

STING mutations could activate downstream signaling in a ligand-independent manner. The hyperactive STING variants were reported in 2011 by Burdette et al. (32) and in 2015 by Tang et al. (59). At the same time, hyperactive STING variants were also found in the patients. For instance, STING-associated vasculopathy with onset in infancy (SAVI) is an autoinflammatory disease driven by gain-of-function mutations in STING, first reported in 2014 (60). In the following several years, many SAVI cases were reported, and the associated mutants are located in multiple sites of the STING protein (61, 62). These mutants could be divided into at least four clusters according to their locations in the STING protein structure (**Figure 7**). The first cluster is localized at the N-terminal domain (NTD) such as H72N variants (60). The second cluster is localized at the dimer interface including V147L/M, F153V, N154S, V155M, and G158A (30, 63–68). The third cluster is located at the helical bundle of LBDα2–α3 and the long LBDβ1–β2 loop outside of the dimer interface including C206Y/G, G207E, R281Q/W, and R284G/S (63, 69–72). The fourth cluster is located at the ligand-binding pocket such as G166E (73). Moreover, SAVI variants that combined two mutations were also found such as S102P/F279L, V155E/L170Q, and L189V/S280R (67, 74, 75). Based on the solved full-length human STING and active STING polymer, the cause of these gain-of-function mutations could be partially explained. For the H72N variant, the substitution of H by N may disrupt the interaction between the N-terminus and the linker of TM2–TM3, leading to the rotation of LBD and activation of STING. Mutations in cluster 2 located in the dimer interface and the substitutions could promote the rotation of LBD breaking the STING inhibition. As for the G166E variant located at the ligand-binding pocket, it could mimic the phosphate group in the cGAMP and interact with R238, resulting in the formation of closed conformation of STING. However, it is challenging to explain mutations in cluster 3 using the present structural model because all these mutations are surface-exposed, and there are no significant changes after ligand binding. Therefore, this site may bind the inhibitory partner or be autoinhibited (70). Ergun et al. proposed that the CTT could bind R281, and their interaction could be impaired by cGAMP binding, supporting the notion of STING autoinhibition by CTT (23). Nevertheless, besides R281, other gain-of-function mutations in this site should be tested to further confirm this viewpoint. In another aspect, STIM1 and TMEM203 were considered as the interaction partners for STING ER residence and they might be bound to this site for STING inhibition (76, 77).

## Conclusion and Future Perspectives

In the past 10 years, the structural study of STING has made huge progress in unveiling the working mechanism of the STING molecule. We now know that STING operates as an obligate domain-swapped dimer. The apo STING adopts an open conformation, and the ligand-binding pocket is located at the crevice formed by the two LBD domains. The structural studies of the STING–CDN complex also show how STING recognizes and differentiates various CDN ligands. The cGAMP binding induces the formation of closed conformation with lid formation covering the ligand in the deep pocket and, at the same time, couples the clockwise 180° rotation of the LBD domain and parallel packing of the array of STING dimer. In addition, the human STING could form a slight bent oligomer in the presence of cGAMP and C53, which might be fit for the anterograde transportation of the STING vesicle. The discovery of a novel agonist binding pocket in the TM domain indicates the existence of diversified activation mechanisms of STING. The head-to-head binding mode of STING and the TBK1 complex reveals that the STING employs a conserved TBM within CTT to plug into the shallow pocket localized between SDD and KD of TBK1. The binding prevents TBK1 from phosphorylating STING in *cis* due to the geometry restraint, while it could only phosphorylate CTT from the adjacent STING dimer, representing a unique *trans* activation mechanism of STING. The full-length structural studies of apo and ligand-bound STING gave a certain explanation for understanding STING-related autoimmune diseases such as SAVI. Moreover, the structural basis of STING could also provide insights into how STING PTM regulates its activation and inhibition.

However, our current knowledge of STING structure and function is still limited. We still do not know how STING, TBK1, and IRF3 assemble into a mega-complex. It is intriguing to know how to reconcile the two controversial activation mechanisms of STING concerning the closed and open conformations induced by cGAMP and diABZI, respectively. We also do not know how STING travels from ER to the Golgi apparatus through COP II transportation machinery. It is interesting to investigate how STING achieves its ER residence *via* interacting with a binding partner such as STIM1 (76). Most importantly, we do not know why STING activation requires 180° rotation of the LBD. The causes of STING-related autoimmune diseases such as SAVI and COPA remain largely elusive (78–81). The structural basis of STING autoinhibition by its CTT should also be considered in future studies. In addition, the structural basis of how the viral protein hijacks the STING for invasion needs further investigation (82, 83). Taken together, much more efforts should be spent to further unravel the working mechanism underlying the cGAS-STING pathway, which is useful for us to develop new therapies to fight infections, autoimmune diseases, and cancers.

## Author Contributions

GS conceived the project. BH, YX, UJ, and GS wrote the manuscript. DL, BY, and CW gave constructive advice and edited the manuscript. All authors contributed to the article and approved the submitted version.

## Funding

This work was supported by grants from the National Key R&D Program of China (2021YFC2301400), the National Natural Science Foundation of China (32070876), the Ministry of Science and Technology of The People's Republic of China (D21004), the International Collaborative Research Project of Shanxi (201903D421054), the Natural Science Foundation for Young Scientists of Shanxi Province (201901D211575), the Science and Technology Major Projects of Shanxi Province (202005D121008), the Shanxi Provincial Science Fund for Distinguished Young Scholars Program (202103021221001), the Shanxi Provincial Key Laboratory of Protein Structure Determination, and the Shanxi Provincial Key Laboratory for Major Infectious Disease Response.

## Conflict of Interest

The authors declare that the research was conducted in the absence of any commercial or financial relationships that could be construed as a potential conflict of interest.

## Publisher’s Note

All claims expressed in this article are solely those of the authors and do not necessarily represent those of their affiliated organizations, or those of the publisher, the editors and the reviewers. Any product that may be evaluated in this article, or claim that may be made by its manufacturer, is not guaranteed or endorsed by the publisher.
